# Supplementary material for: NECTIN4: A Novel Therapeutic Target for Melanoma
Source: Int J Mol Sci. 2021 Jan 19;22(2):976. doi: 10.3390/ijms22020976 (PMC7835875; doi:10.3390/ijms22020976)
Supplement: Supplementary file 1 [file ijms-22-00976-s001.pdf]

Supplementary

NECTIN4: A Novel Therapeutic Target for Melanoma

Yuka Tanaka [1,†], Maho Murata [1,†], Che-Hung Shen [2], Masutaka Furue [1,3] and Takamichi Ito [1,*]

[1] Department of Dermatology, Graduate School of Medical Sciences, Kyushu University,
Fukuoka 812-8582, Japan; yukat53@med.kyushu-u.ac.jp (Y.T.); muratama@dermatol.med.kyushu-u.ac.jp (M.M.); furue@dermatol.med.kyushu-u.ac.jp (M.F.)

[2] National Institute of Cancer Research, National Health Research Institutes, Tainan 70456, Taiwan;
chshen@nhri.org.tw

[3] Research and Clinical Center for Yusho and Dioxin, Kyushu University Hospital, Fukuoka 812-8582, Japan

* Correspondence: takamiti@dermatol.med.kyushu-u.ac.jp; Tel.: +81-92-642-5585

† These authors contributed equally to this work.

This file contains:

Supplementary Figures S1-S6

Supplementary Figure S1

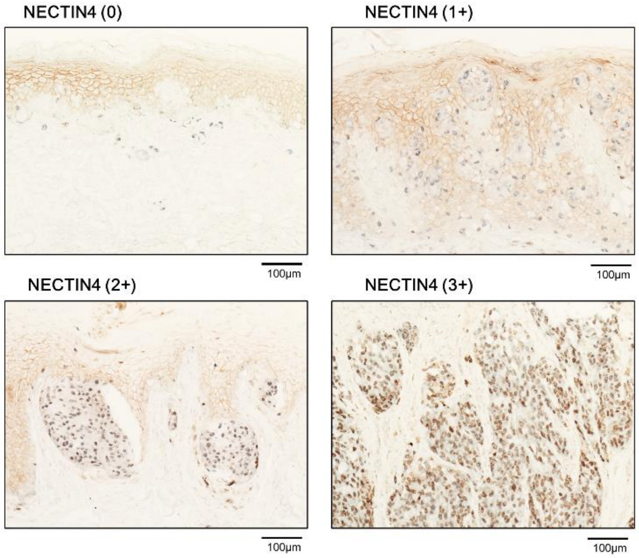

Supplementary Figure S1. NECTIN4 and SOX10 expression in melanoma tissues. Immuno-double staining of NECTIN4 and SOX10 (a melanoma specific marker) in patients' tumor tissues. Representative images from 126 melanoma samples are shown. NECTIN4 (membranous and nuclear staining in brown) and SOX10 (nuclear staining in blue) were stained on the same sample. Counter nuclear staining with hematoxylin was not performed in these sections. Scale bar = 100 μm.

Supplementary Figure S2

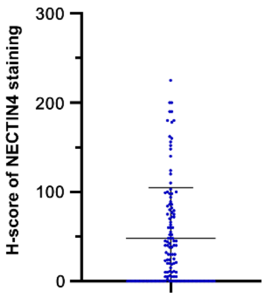

Supplementary Figure S2. H-score of NECTIN4 staining.
Immunohistochemical results of NECTIN4 staining were evaluated by the H-score system, a semiquantitative approach. H-scores of 126 malignant melanoma patients are shown (n = 126).

Supplementary Figure S3

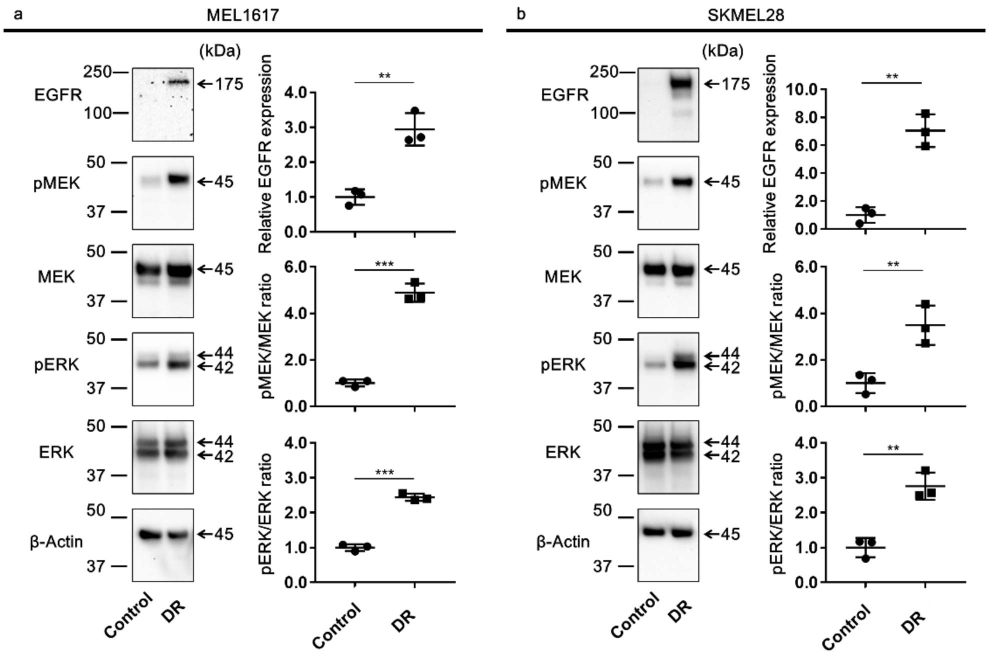

Supplementary Figure S3. EGFR and MEK/ERK signal is activated in dabrafenib-resistant cell lines.

Relative EGFR protein expression, pMEK/MEK ratio, and pERK/ERK ratio in (**a**) MEL1617 and (**b**) SKMEL28, and their DR cells cultured with 1 μM dabrafenib. Representative blot images (left panels), relative EGFR expression, pMEK/MEK, and pERK/ERK ratio (right panels, n =3) are shown. Data are presented as the mean ± standard deviation of three independent experiments. β-Actin served as a loading control. ***P* < 0.01 and ****P* < 0.001 determined by Student's unpaired two-tailed *t*-test.

Supplementary Figure S4

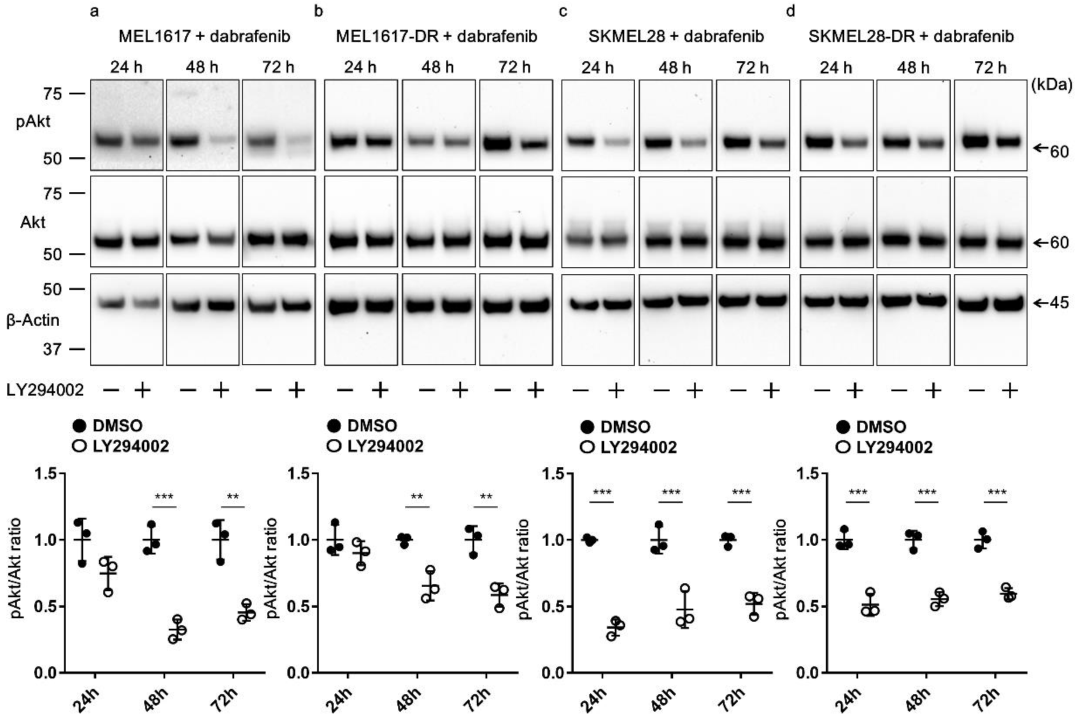

Supplementary Figure S4. pAkt/Akt ratio in melanoma cell lines under the treatment with LY294002.

(**a**) MEL1617, (**b**) MEL1617-DR, (**c**) SKMEL28, and (**d**) SKMEL28-DR cell lines were treated with DMSO (0.1%) or LY294002 (10 μM) in the presence of dabrafenib for 24–72 h and assessed for pAkt/Akt ratio by western blot (n = 3). Representative blot images (upper panels) and pAkt/Akt ratio (lower panels) are shown. Data are presented as the mean ± standard deviation of three independent experiments. β-Actin served as a loading control. $^{**}P < 0.01$ and $^{***}P < 0.001$ determined by Student's unpaired two-tailed *t*-test.

Supplementary Figure S5

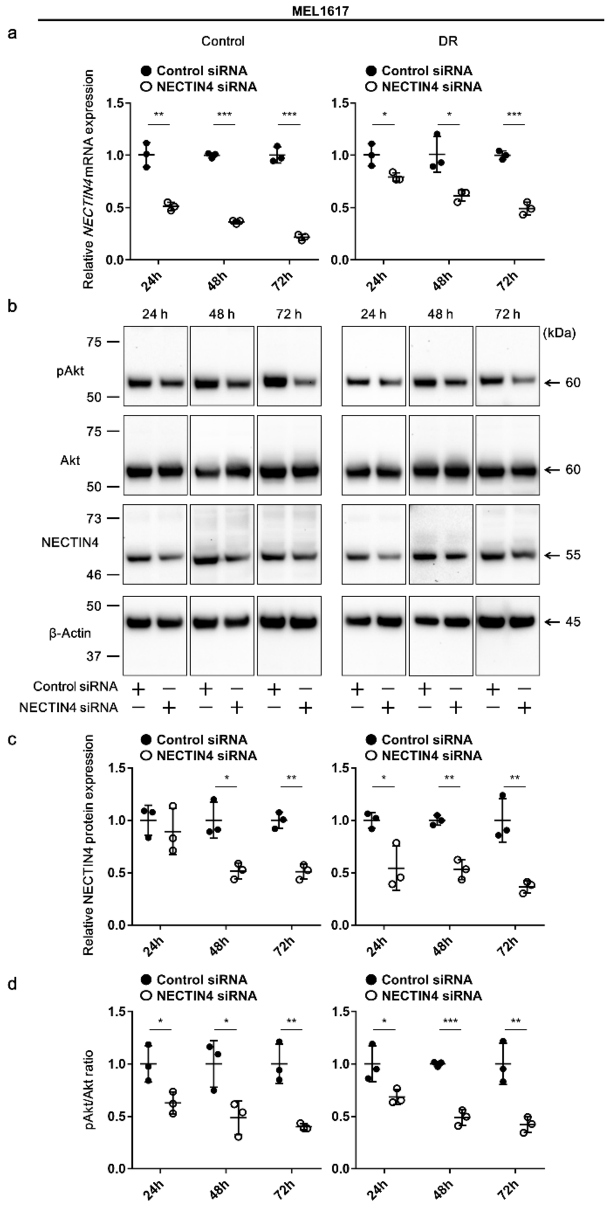

Supplementary Figure S5. Knockdown efficiency of NECTIN4 and pAkt/Akt ratio during 24–72 h after transfection in MEL1617 and MEL1617-DR cells.

Cells were transfected with control or NECTIN4 siRNA for 24–72 h and assessed for NECTIN4 knockdown efficiency and pAkt/Akt ratio. (**a**) Relative *NECTIN4* mRNA expression (n = 3), (**b**) representative blot images, (**c**) relative NECTIN4/β-Actin ratio (n = 3), and (**d**) pAkt/Akt ratio (n = 3) in NECTIN4 siRNA transfected MEL1617 (left panels) and MEL1617-DR (right panels) is shown. Data are presented as the mean ± standard deviation of three independent experiments. β-Actin served as a loading control. *$P < 0.05$, **$P < 0.01$, and ***$P < 0.001$ determined by Student's unpaired two-tailed *t*-test.

Supplementary Figure S6

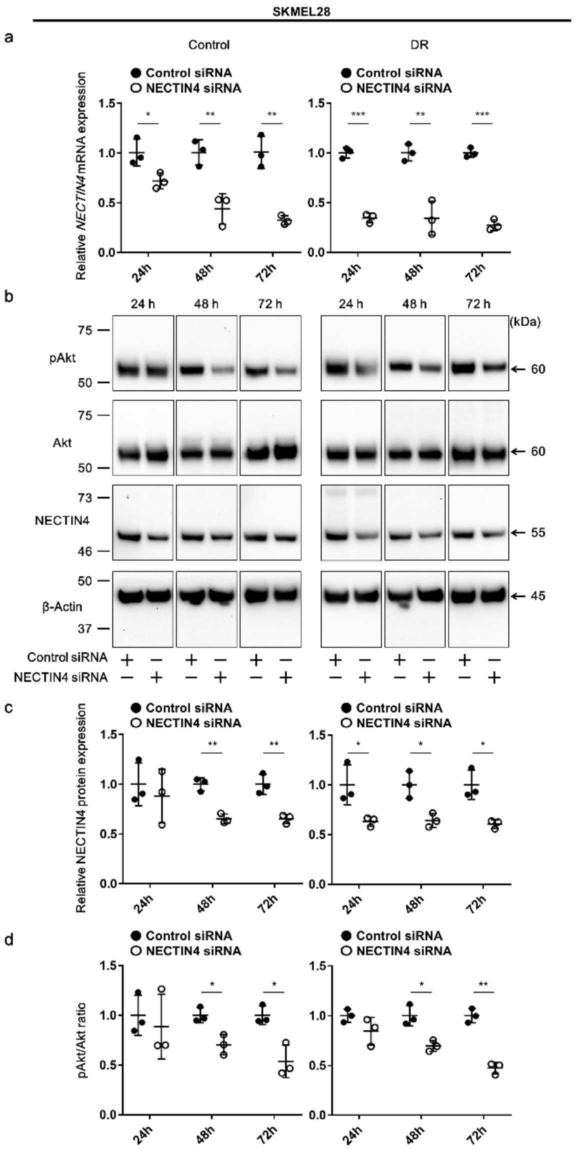

Supplementary Figure S6. Knockdown efficiency of NECTIN4 and pAkt/Akt ratio during 24–72 h after transfection in SKMEL28 and SKMEL28-DR cells.

Cells were transfected with control or NECTIN4 siRNA for 24–72 h and assessed for NECTIN4 knockdown efficiency and pAkt/Akt ratio. (**a**) Relative *NECTIN4* mRNA expression (n = 3), (**b**) representative blot images, (**c**) relative NECTIN4/β-Actin ratio (n = 3), and (**d**) pAkt/Akt ratio (n = 3) in NECTIN4 siRNA transfected SKMEL28 (left panels) and SKMEL28-DR (right panels) is shown. Experiments were independently repeated three times. Data are presented as the mean ± standard deviation of three independent experiments. β-Actin served as a loading control. $^{*}P < 0.05$, $^{**}P < 0.01$, and $^{***}P < 0.001$ determined by Student's unpaired two-tailed *t*-test.
